# Characterization of immunomodulatory responses induced by manuka honey

**DOI:** 10.3389/fimmu.2022.1020574

**Published:** 2022-11-02

**Authors:** Razan J. Masad, Rasha A. Nasser, Ghada Bashir, Yassir A. Mohamed, Ashraf Al-Sbiei, Besan H. Al-Saafeen, Maria J. Fernandez-Cabezudo, Basel K. Al-Ramadi

**Affiliations:** ^1^ Department of Medical Microbiology and Immunology, College of Medicine and Health Sciences, United Arab Emirates University, Al Ain, United Arab Emirates; ^2^ Department of Biochemistry and Molecular Biology, College of Medicine and Health Sciences, United Arab Emirates University, Al Ain, United Arab Emirates; ^3^ Zayed Center for Health Sciences, United Arab Emirates University, Al Ain, United Arab Emirates

**Keywords:** manuka honey, TNF-α, inflammatory response, immunomodulation, neutrophil recruitment

## Abstract

Manuka honey (MH) is known for its wound-healing, anti-microbial, anti-oxidant and anti-tumor properties. However, there is conflicting evidence regarding the role of MH in inflammatory responses, with some studies highlighting its pro-inflammatory capacity and others showing that it has a predominantly anti-inflammatory activity. The current study is aimed at characterizing the immunomodulatory capacity of MH using both *in vitro* and *in vivo* approaches, focusing on the underlying mechanisms. Treatment of RAW 264.7 macrophages with 1% MH (w/v) resulted in a significant increase in the gene expression (~26-fold) and secretion (~27-fold) of tumor necrosis factor-alpha (TNF-α). Similarly, an increase was observed in the gene expression of other inflammatory cytokines including interleukin-1β (*IL-1β)*, interleukin-6 (*IL-6*), and inducible nitric oxide synthase (i*NOS*), as well as the chemokines; (C-X-C motif) ligand 2 (*CXCL2*) and (C-C) motif ligand 2 (*CCL2*). Using an *in vivo* model, intraperitoneal (i.p.) administration of MH in C57BL/6 mice elicited a peritoneal response characterized by a significant expansion in the number of peritoneal exudate cells (PECs), which was mainly due to a 35-fold increase in the recruitment of neutrophils. Importantly, this response was evident in toll-like receptor 4 (TLR4)-defective C3H/HeJ mice, indicating that the observed stimulatory effect occurs independently of TLR4 and unlikely to be mediated by any lipopolysaccharide (LPS) contaminant. MH administration also led to changes in the phenotypic expression and functional maturation of peritoneal macrophages, as evidenced by a shift towards the CD11b^lo^ F4/80^lo^ phenotype and an increase in the expression of major histocompatibility complex (MHC) class II proteins. In contrast, the MH-initiated peritoneal response was largely abrogated in mice deficient in myeloid differentiation primary response 88 (MyD88) protein, a critical adaptor of most TLR signaling pathways. Thus, the current findings help to characterize the immunostimulatory properties of MH and their dependence on TLR signaling, and highlight the potential utility of MH as an immunomodulatory agent in a variety of disorders.

## Introduction

Honey is an effective natural medicine for a wide range of disorders. Among the well-studied honey types, Manuka honey (MH) has been shown to have numerous therapeutic properties. MH is produced by the European honeybee *Apis mellifera* which forages on the Manuka tree (*Leptospermum scoparium*) that grows throughout New Zealand and southeastern Australia (1). In the mid-1980s, the antimicrobial activity of MH against different bacterial species was reported. However, the specific active ingredients responsible for this activity remained unclear for many years. In 2008, these ingredients were revealed after the identification of methylglyoxal (MGO), which is produced by the spontaneous dehydration of its precursor dihydroxyacetone, a naturally occurring phytochemical found in the nectar of Leptospermum flowers (2–4). In addition to MGO and dihydroxyacetone, leptosperin, a glycoside found in the Leptospermum honey, has been also suggested to modulate the activity of MH (5). In fact, the Unique Manuka Factor (UMF) grading system appraises the purity and quality of MH according to the presence of these three natural components (6).

There is extensive evidence demonstrating beneficial activities for MH, including wound-healing, anti-microbial, anti-oxidant and anti-cancer properties, as recently reviewed (6–8). However, there is conflicting data regarding the role of MH in inflammation. Some studies demonstrated the capacity of MH to induce pro-inflammatory cytokines, including tumor necrosis factor-alpha (TNF-α), interleukin-1β (IL-1β), and interleukin-6 (IL-6), by macrophages (9–11). Other studies, however, affirmed that MH acts mainly as an anti-inflammatory agent, counteracting the effect of lipopolysaccharide (LPS) on macrophages (12), and inhibiting the production of TNF-α by neutrophils (13). Additionally, MH treatment was found to significantly reduce the neutrophil superoxide release (14, 15).

The role of MH as an immunomodulatory agent was also investigated in preclinical models. In one study, a 7 day-pretreatment with MH ameliorated tissue damage in an acute, ethanol-induced, gastric ulcer model (16). This was correlated with increased levels of mucosal antioxidants and a reduction in plasma levels of several pro-inflammatory cytokines, including TNF-α, IL-1β, and IL-6. On the other hand, oral administration of MH to breast cancer-bearing Sprague Dawley rats resulted in a significant reduction in tumor growth, which was accompanied by an increase in the serum level of the pro-inflammatory cytokine interferon-gamma (IFN-*γ*), indicating an immunomodulatory anti-tumor effect of MH (17).

The current work aimed to further elucidate the immunomodulatory role of MH using both *in vitro* and *in vivo* approaches. Our findings provide evidence for a pro-inflammatory role of MH, and highlight its potential as an immunostimulatory agent for a variety of disorders.

## Materials and methods

### Honey

Manuka honey (UMF^®^ 20+ from ApiHealth, Auckland, New Zealand) was used in the study, and diluted in sterile PBS or DMEM media under aseptic conditions. As a control for MH, a sugar solution, designated sugar control (SC), containing equivalent concentrations of the three major sugars in honey (38.2% fructose, 31.3% glucose, and 1.3% sucrose) was used (18). SC and MH concentrations are expressed as % w/v. For *in vitro* studies, appropriate dilutions to the desired concentrations were made fresh in culture medium, filtered through 0.2 μm polyethersulfone (PES) syringe filter (Corning, Glendale, AZ, USA) before addition to the cells in culture. For *in vivo* studies, fresh reagents were prepared on the day of use in saline, filtered through 0.2 μm PES syringe filter (Corning, Glendale, AZ, USA) and used for intraperitoneal (i.p.) administration.

### Cell lines and mice

The murine RAW264.7 macrophage cell line was provided by Dr. Jamil Azzi (Brigham and Women’s Hospital, Harvard University, Boston, USA). The cells were maintained in DMEM media supplemented with 10% FBS, antibiotics (penicillin 100 IU/ml; streptomycin 100 IU/ml), and gentamycin (50 µg/ml) (Gibco-ThermoFisher Scientific, Waltham, MA, USA). Cells were incubated at 37°C in a humidified atmosphere of 5% CO_2_ and used when in the log phase of growth. C57BL/6 mice were purchased from the Jackson Laboratory. MyD88-deficient (MyD88^-/-^) mice were provided by Dr Richard Flavell (Department of Immunobiology, Yale University School of Medicine, USA) and have been previously described (19). C3H/HeJ mice, purchased from Harlan Olac (Bicester, UK), carry a mutated allele of the TLR4 gene (TLR4^d^) and thus are hyporesponsive to LPS (20). All animals were bred in the animal facility of the College of Medicine and Health Sciences, UAE University. For the current study, male mice at the age of 8–10 weeks were used. Mice received rodent chow and water ad libitum. All studies involving animals were carried out in accordance with, and after approval of, the Animal Research Ethics Committee of the United Arab Emirates University (Protocols #A12-13 and ERA-2019-5853).

### RAW 264.7 macrophage culture and treatments

RAW 264.7 cells were seeded in a 6-well plate (1.5x10^6^ cells/well) overnight in DMEM media supplemented with 2% FBS, antibiotics (penicillin 100 IU/ml, streptomycin 100 IU/ml), and gentamycin (50 µg/ml) (Gibco-ThermoFisher Scientific). Following overnight seeding, cells were either left untreated or treated for 2-24 hours with a solution of 1% SC or 1% MH, or with 1μg/ml LPS (*E.coli* 0111:B4, Sigma Aldrich, St Louis, MO, USA) as a positive control. All treatments were prepared in 5% FBS-DMEM medium. At the end of the incubation period, cell-free culture supernatants were collected and the levels of nitric oxide (NO), and TNF-α and IL-1β cytokines were determined. Accumulation of nitrite ions was used to determine the production of NO according to the Griess method, as described previously (21). TNF-α and IL-1β levels were determined using specific ELISA kits (BD Biosciences, CA, USA; cat#555268 for TNF-α and cat#559603 for IL-1β). Cells were also collected from each well, pelleted and suspended in trizol for gene expression analysis by real-time PCR.

### Quantitative RT-PCR

qRT-PCR was carried out as previously detailed (22). RAW 264.7 cells using Trizol reagent (Thermo Fisher Scientific) and RNA easy mini kit (Qiagen, Germany). The quality and quantity of RNA were determined using the Nanodrop ND-1000 spectrophotometer (Thermo Fisher Scientific, Waltham, MA). For cDNA synthesis, 2.0 ug of total RNA was reverse transcribed in a 50 ul reaction using Taqman reverse transcription reagents (Applied Biosystems, Foster City, Ca, USA) as per manufacturer’s instructions. Real time qRT-PCR was performed using QuantStudio™ 7 flex real-time PCR system with master mix reagents and premade TaqMan primers and probes for the following genes; *TNF-α*, [F: 5′-CCT CCC TCT CAT CAG TTC TAT-3′, R: 5′-CTA GTT GGT TGT CTT TGA GAT CC-3′, Probe: 5′-6-FAM-ACA AGC CTG TAG CCC ACG TCG TAG-BHQ-1-3′] (Metabion, Steinkirchen, Germany); Mm00443260_g1; *IL-1β*, Mm00434228_m1; *IL-6*, Mm00446190_m1; *iNOS*, Mm00440502_m1; *CXCL2*, Mm00436450_m1; *CXCL10*, Mm99999072_m1; and *MCP-1* (*CCL2*), Mm00441242_m1(All from Applied Biosystems). The mRNA levels of target genes were normalized according to the comparative ΔCq method to respective mRNA levels of the housekeeping gene HPRT (Mm01545399_m1)(Applied Biosystems). The expression of the target gene is reported as the level of expression relative to HPRT.

### *In vivo* studies

Mice were injected i.p. with 0.5 ml of a saline solution containing 50% MH or 50% SC. Based on our previous studies, this dilution is suitable for parenteral administration of MH (23). Forty eight hours later, mice were euthanized by exposure to isoflurane, and peritoneal exudate cells (PECs) were collected as previously described (24). Briefly, PECs were harvested by peritoneal lavage using cold PBS and spun down. The pelleted cells were then resuspended in 1 ml PBS, counted using the trypan blue exclusion method, and processed for flow cytometry analysis.

### Flow cytometry

Peritoneal cells were stained for multi-color flow cytometry analysis following standard procedures (25). Briefly, 1.0 x10^6^ cells were dispensed into the wells of a 96-well round-bottom plate. Cells were pre-incubated with anti-CD16/CD32–specific mAb (101320/93, BioLegend, San Diego, CA) for 30 minutes at 4°C to block FcγRIII/II receptors, and prevent any non-specific binding of the antibodies. Cells were then incubated with Zombie Aqua viability dye to exclude non-viable cells. Following washing, cells were stained with a mixture of fluorochrome-conjugated monoclonal antibodies to immune cells surface markers (all purchased from BioLegend) for 30 minutes at 4^0^C. For the C57BL/6 and MyD88^-/-^ mouse strains, the phenotype panel included the following antibodies: CD3-BV786 (catalog/clone100232/17A2), CD11b-Alexa Fluor 488 (catalog/clone 101217/M1/70), CD19-PE-Dazzle 594 (catalog/clone 115554/6D5), and NK1.1-PE-A (catalog/clone 108708/PK136). For the C3H/HeJ mouse strain, the phenotype panel included the following antibodies: CD3-BV786 (catalog/clone100232/17A2), CD11b-Alexa Fluor 488 (catalog/clone 101217/M1/70), CD19-PE-A (catalog/clone 115508/6D5), and CD49b-PE-Dazzle 594 (catalog/clone 108924/Dx5).

Analysis of myeloid cell subpopulations was carried out using a second panel of mAbs. For all tested mouse strains, the myeloid panel included the following antibodies: CD11b-Alexa Flour 488 (catalog/clone (101217/M1/70), CD19-PE-Dazzle 594 (catalog/clone115554/6D5), CD11c-APC-A (catalog/clone 117309/N418), Ly6G-BV605 (catalog/clone 127639/1A8), F4/80-BV421(catalog/clone 123137/BM8), and MHC class II- APC-Cy7 (catalog/clone 107628/M5/114.15.2). This MHC class II-specific mAb reacts with a polymorphic epitope shared by many haplotypes, including I-A^b^ and I-E^k^ MHC class II alloantigens. Data were collected on 20,000 cells using a FACSCelesta flow cytometer (BD Biosciences) and analyzed using FlowJo software (BD Biosciences).

### Statistical analysis

All statistical analyses were performed using GraphPad Prism 9.0 (GraphPad, San Diego California, USA). Statistical significance between control and treated groups was determined using the unpaired Student’s t-test (two-tailed). In all analyses, *p ≤ *0.05 was considered statistically significant (* p ≤ 0.05, ** p ≤ 0.01, *** p ≤ 0.001, **** p ≤ 0.0001).

## Results

### Manuka honey induces key inflammatory cytokines and chemokines in macrophages

To elucidate the potential immunomodulatory effects of MH, we first investigated the consequences of exposing RAW 264.7 macrophages to a low concentration of MH (1% w/v) for 2, 6 or 24 hrs. Based on our previous studies, this concentration is not toxic to normal cells and does not induce any significant apoptotic or necrotic effects (18, 26). LPS, a major component of the outer membrane of Gram-negative bacteria and a potent inducer of inflammatory responses in macrophages (27, 28) was used as a positive control. As a negative control, cells were either left untreated or treated with an equivalent concentration of sugar control solution (1% SC).

**Figure 1:**
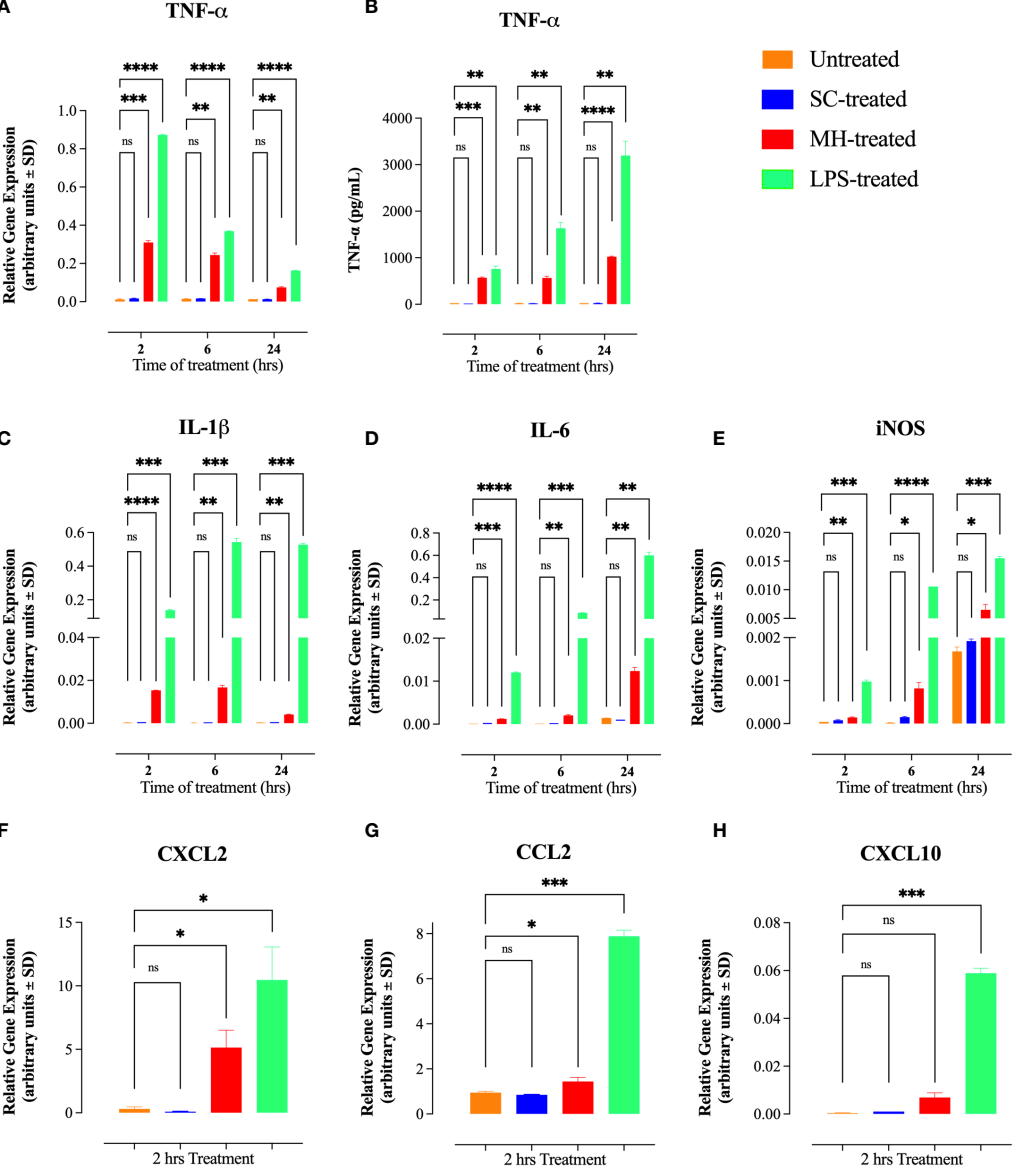
MH treatment induces inflammatory cytokines and chemokines in RAW 264.7 macrophages. **(A, B)** mRNA expression **(A)** and protein level **(B)** of TNF-*α* following 2, 6 and 24 hours of treatment with SC, MH or LPS. **(C-E)** Gene expression analysis of IL-1β **(C)**, IL-6 **(D)**, and iNOS **(E)** following 2, 6, or 24 hours of the indicated treatment. **(F-H)** mRNA expression of CXCL2 **(F)**, CCL2 **(G)**, and CXCL10 **(H)** chemokines following 2 hours of treatment. As a negative control, cells were left untreated. LPS was used as a positive control. The data are expressed as means ± SD of 2 replicates per group and are representative of 2 independent experiments. Asterisks denote statistically significant differences between the indicated experimental group and the untreated group. *p* values were calculated using the unpaired Student’s t-test (ns; p > 0.05, * p ≤ 0.05, ** p ≤ 0.01, ***p≤ 0.001, **** p ≤ 0.0001).

Following treatment for various time periods, the level of expression of several key inflammatory cytokines and chemokines was determined by qRT-PCR. Treatment of RAW 264.7 cells with 1% MH resulted in a dramatic, 26-fold, upregulation in the expression of *TNF-α* within 2 hrs (**Figure 1A**). The upregulated *TNF-α* gene expression was also evident at the 6 and 24 hrs time points. The magnitude of the MH-induced *TNF-α* response is substantial, especially when compared with the LPS-induced responses (**Figure 1A**). Moreover, the upregulation in *TNF-α* expression was confirmed at the protein level in cell-free culture supernatants by ELISA, where ~ 27-fold increase in TNF-α content was evident as early as 2 hrs after culture and continued to be observed thereafter at the 6 and 24 hrs time points (**Figure 1B**). We also observed a small, but significant, upregulation in the expression of other inflammatory genes, including *IL-1β*, *IL-6* and *iNOS* (**Figures 1C–E**). However, these responses were qualitatively small in magnitude and did not result in any observable increase in the protein content for IL-1β cytokine or nitrite ion accumulation, a marker of iNOS enzymatic function (Data not shown). In addition to these inflammatory mediators, we also tested for MH-induced changes in chemokine expression by RAW 264.7 macrophages. Two of the chemokine families known to be secreted by these cells are monocyte chemoattractant proteins (e.g. MCP-1 or CCL2) and macrophage inflammatory proteins (MIP; e.g. CXCL2). Both of these inflammatory chemokines are potent chemoattractants that control the recruitment of polymorphonuclear leukocytes in inflammation and tissue injury (29). Gene expression analysis demonstrated the ability of MH to also induce *CXCL2* and *CCL2* chemokines (**Figures 1F, G**). In contrast, there was no evidence for the induction of the chemokine *CXCL10* gene (also known as interferon gamma-induced protein 10; IP-10), which is secreted in response to IFN-γ and preferentially regulates recruitment of inflammatory T lymphocytes (30) (**Figure 1H**). It is worth noting that treatment with 1% SC induced only baseline levels of the tested cytokines and chemokines, which were indistinguishable from untreated cell cultures.

**Figure 2:**
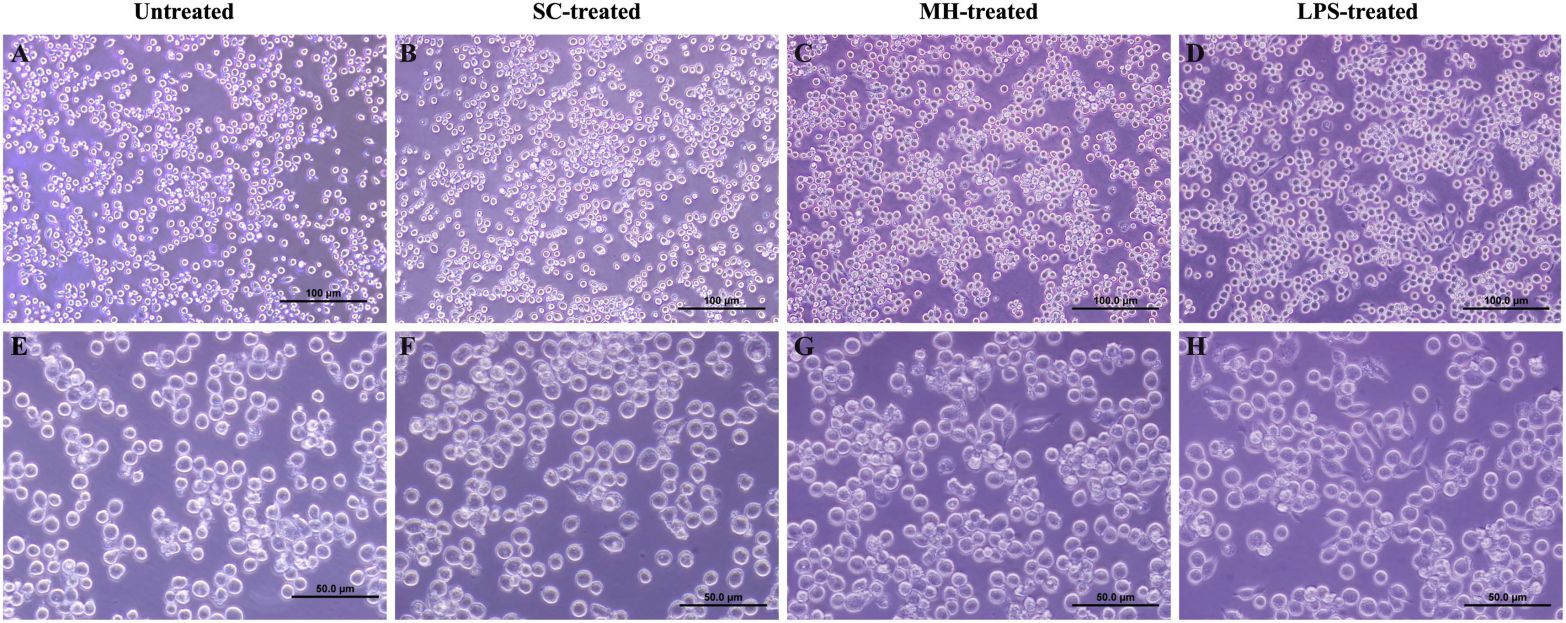
MH-treatment induces changes in the morphology of RAW264.7 cells. Morphology of RAW264.7 cells following 2 hours incubation in DMEM media (untreated cells) **(A, E)** and 2 hours treatment with SC **(B, F)**, MH **(C, G)**, and LPS **(D, H)**. Images were taken at 20X (Scale bar 100 μm) and 40X magnification (Scale bar 50 μm).

Of note, similar to LPS-treated cells, MH-treatment stimulated the formation of spindle-shaped pseudopodia within 2 hours of culture, which is indicative of cell activation. On the other hand, the untreated and sugar control-treated cells maintained their original round shape (**Figure 2**).

### Manuka honey induces changes in the peritoneal cavity of C57BL/6 mice

**Figure 3:**
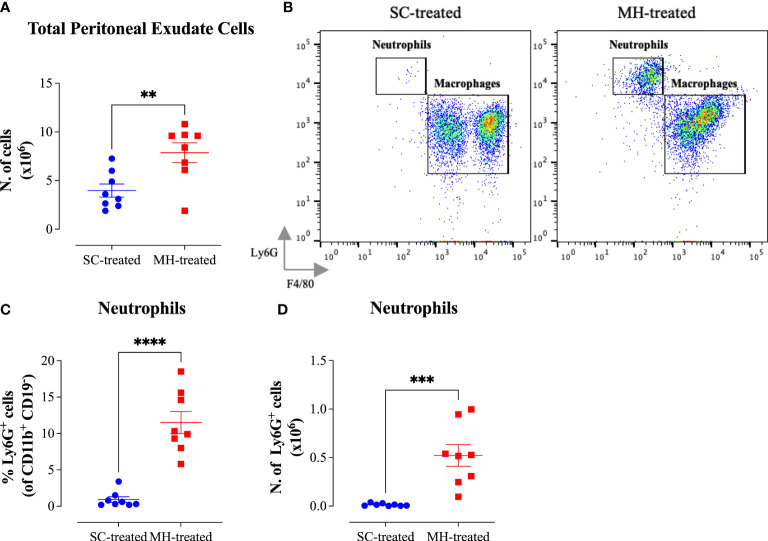
MH induces the recruitment of neutrophils into the peritoneal cavity of C57BL/6 mice. **(A)** Total number of PECs following treatment with SC or MH. **(B)** Representative dot plots showing Ly6G^+^ neutrophils and F4/80^+^ macrophage subpopulations within the PECs (gated on CD11b^+^ CD19^-^ CD11c^-^ cells). **(C, D)** Quantification of the percentage **(C)** and absolute number **(D)** of neutrophils (Ly6G^+^ cells) in the peritoneal cavity of C57BL/6 mice following treatment with SC or MH. Asterisks denote statistically significant differences between the MH-treated and SC-treated groups. The values for individual mice in a group (and mean ± SEM) are shown (SC-treated: n=8, MH-treated: n=8), pooled from 2 individual experiments. p values were calculated using the unpaired Student’s t-test (**p≤ 0.01, *** p≤ 0.001, **** p≤ 0.0001).

The potential of MH to modulate systemic immunity was next investigated. Cellular alterations in the peritoneal cavity were studied following i.p. administration of MH or SC solutions to C57BL/6 mice. MH administration induced a doubling in the number of peritoneal exudate cells (PECs) compared to the group injected with the SC solution (**Figure 3A**). Multi-color flow cytometry was utilized to analyze the cellular changes in the peritoneal cavity following MH administration in more detail. The gating strategy employed to analyze the different cell populations is shown in **Supplementary Figure 1** (**sFigure 1**).

The results of this analysis revealed that while the percentage of total myeloid (CD11b^+^ CD19^-^) cells, which constitute the largest population within the PECs (>50%), increased only slightly in MH-treated mice compared to the SC-treated group (**Supplementary Figures 2A**), the absolute number of these cells was significantly increased (2-fold) following MH treatment (**Supplementary Figures 2H**). This was largely accounted for by the large increase in both the percentage (12.6-fold) and absolute number (35.4-fold) of neutrophils (Ly6G^+^ CD11b^+^) following MH treatment (**Figures 3B–D**). Regarding other myeloid cell subpopulations, there was no difference in the percentage or absolute number of macrophages (CD11b^+^ F4/80^+^) following MH treatment (**Supplementary Figures 2B, I**). On the other hand, a small but significant increase in both the percentage (~1.5-fold) and absolute number (2.7-fold) of dendritic cells (CD11b^+^ CD11c^+^) was observed (**Supplementary Figures 2C, J**). Nevertheless, these cells still constituted a minor population (~6.7%) of the total PECs. In terms of lymphoid cell subpopulations, there was no major change in the % of CD3^+^ T cells or B2 cells (CD19^+^ CD11b^-^) after MH treatment (**Supplementary Figures 2D, F**). Moreover, while the percentage of B1 cells (CD19^+^ CD11b^+^) significantly decreased by 2.5-fold following MH treatment, the absolute number of these cells remained unchanged (**Supplementary Figures 2E, L**). This alteration is most likely due to the increased infiltration by neutrophils as a result of MH administration. Finally, a small but significant increase in both the percentage and absolute number of NK cells (NK 1.1^+^) was observed following MH treatment (**Supplementary Figures 2G, N**). Taken together, these results suggest that MH can induce changes in the PECs largely through the recruitment of pro-inflammatory cells.

**Figure 4:**
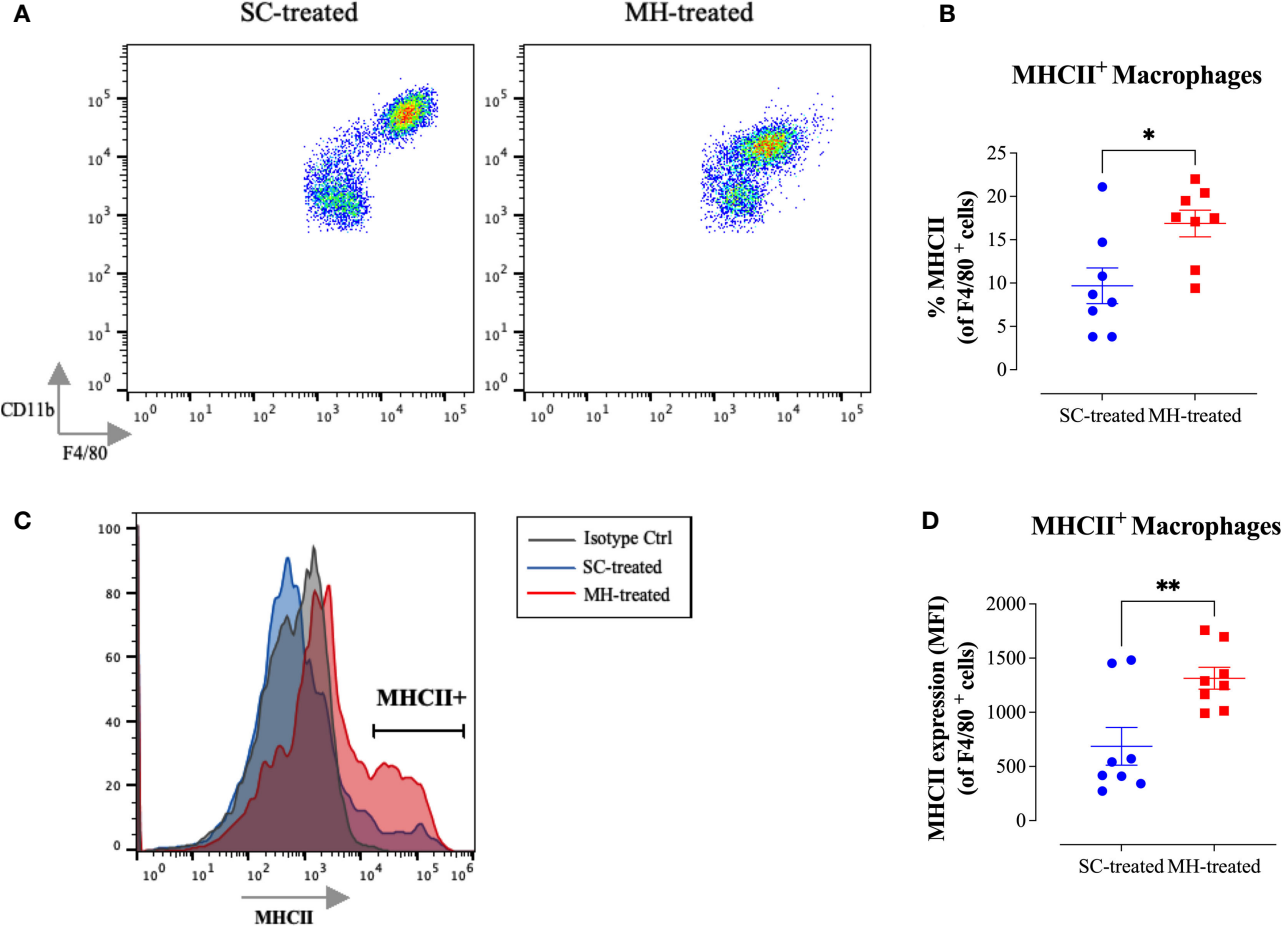
MH induces changes in the maturation of peritoneal myeloid cells in C57BL/6 mice. **(A)** Representative dot plots showing F4/80^+^ CD11b^+^ cells (gated on macrophages) in SC-treated and MH-treated mice. **(B)** Representative overlay histograms showing MHC class II expression on F4/80^+^ macrophages of SC-treated and MH-treated mice. Grey histogram indicates staining with isotype matched control antibody. **(C, D)** Quantification of the percentage **(C)** and median fluorescence intensity **(D)** of MHC class II^+^ macrophages in SC-treated and MH-treated groups. Asterisks denote statistically significant differences between the MH-treated and SC-treated groups. The values for individual mice (mean ± SEM) are shown (SC-treated: n=8, MH-treated: n=8), pooled from 2 independent experiments. p values were calculated using the unpaired Student’s t-test (*p ≤ 0.05, **p ≤ 0.01).

Further analysis of the peritoneal macrophage subpopulation (**Figure 3B**) highlighted some changes in their expression pattern of CD11b and F4/80 cell surface markers. In control mice, two different macrophage populations could be discerned (**Figure 4A**; SC-treated group), a population expressing high levels of both markers (F4/80^hi^ CD11b^hi^ macrophages) and another population that expressed low levels of these markers (F4/80^lo^ CD11b^lo^). In MH-treated mice, PEC macrophages appeared to lose their expression of both markers, with the majority becoming F4/80^lo^ CD11b^lo^ cells (**Figure 4A**). Furthermore, the functional activity of these cells was qualitatively altered, as evidenced by a significant increase in the percentage of macrophages expressing MHC class II molecules (**Figures 4B, C**). This was further confirmed by demonstrating a significant increase in the expression level (median fluorescence intensity; MFI) of MHC class II proteins among PEC macrophages following MH administration (**Figure 4D**). These findings suggest that, in addition to the recruitment of neutrophils, i.p. administration of MH induced the functional maturation of PEC macrophages and their antigen-presenting capacity.

**Figure 5:**
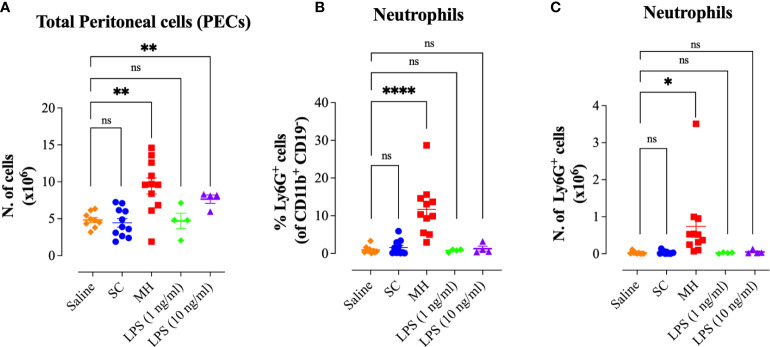
MH-induced peritoneal immune response is independent of its LPS content. **(A)** Total number of PECs following treatment with saline, 50% SC, 50% MH, 1 ng/ml LPS and 10 ng/ml LPS **(B, C)** Quantification of the percentage **(B)** and absolute number **(C)** of neutrophils (Ly6G^+^ cells) in the peritoneal cavity of C57BL/6 mice following treatment with saline, 50% SC, 50% MH, LPS (1 ng/ml) and LPS (10 ng/ml). Asterisks denote statistically significant differences between the indicated experimental group and the saline-treated group.. The values for individual mice in a group (and mean ± SEM) are shown (Saline: n= 9, SC-treated: n=11; MH-treated: n=11, LPS (1 ng/ml): n= 4, LPS (10 ng/ml): n=4), pooled from 3 individual experiments. *p* values were calculated using the unpaired Student’s t-test (ns; p > 0.05, *p ≤ 0.05, **p ≤ 0.01, ****p ≤ 0.0001).

We next compared the peritoneal immune response observed after administration of 50% SC solution or saline. The data confirmed that administration of saline or 50% SC solution failed to induce any cell recruitment into the peritoneal cavity (**Figures 5A–C**). These findings indicate that the peritoneal response triggered by MH is independent of its major sugar component. Next, we sought to investigate whether the MH-induced peritoneal cellular response could be due to LPS contaminants of MH. Although the LPS content in MH is typically very low (<0.3 ng/ml) (10), it was important to demonstrate whether the observed *in vivo* responses are due to the presence of LPS in MH. To this end, we investigated the cellular response after i.p. administration of LPS using either 0.5 ng (equivalent to the reported LPS content in MH) or 5 ng per dose, approximately 10-fold higher than its content in MH. There was no discernable response observed following injection of 0.5 ng LPS (**Figures 5A–C**). When LPS was administered at the higher dose (5 ng), a small but significant increase (1.6-fold) in the number of total PECs was observed (**Figure 5A**). However, there was no evidence of neutrophil recruitment into the peritoneal cavity (**Figures 5B, C**). In sharp contrast, a substantial increase in the PEC number and cellular infiltration was observed following MH administration (**Figures 5A–C**). These findings indicate that the observed MH-induced peritoneal response is independent of any potential LPS content.

### Requirements for MH-initiated immunomodulatory response

**Figure 6:**
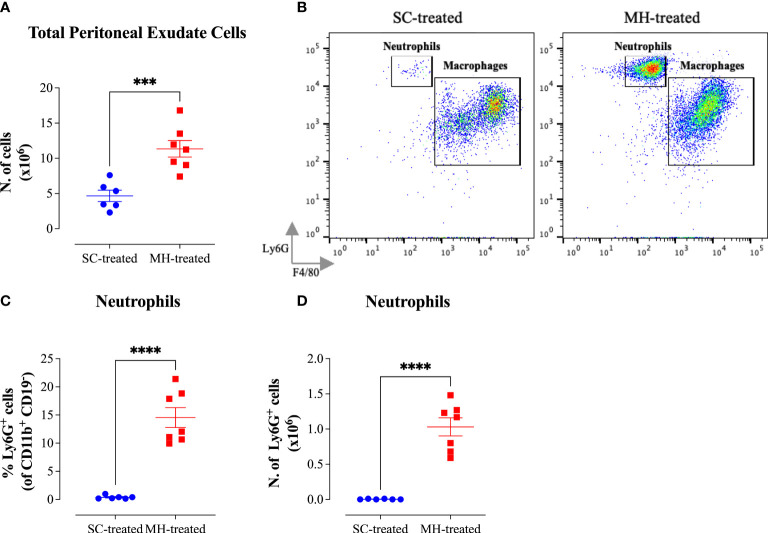
Cellular alterations in the peritoneal cavity following MH administration in C3H/HeJ mice. **(A)** Total number of PECs following treatment with SC or MH. **(B)** Representative dot plots showing Ly6G^+^ neutrophils and F4/80^+^ macrophages within the PECs (gated on CD11b^+^ CD19^-^ CD11c^-^ cells). **(C, D)** Quantification of the percentage **(C)** and absolute number **(D)** of neutrophils (Ly6G^+^ cells) in the peritoneal cavity of C3H/HeJ mice following treatment with SC or MH. Asterisks denote statistically significant differences between the MH-treated group and the SC-treated group. Asterisks denote statistically significant differences between the MH-treated group and SC-treated groups. The values for individual mice in a group ± SEM are shown (SC-treated: n=6; MH-treated: n=7), pooled from 2 individual experiments. *p* values were calculated using the unpaired Student’s t-test (***p ≤0.001, ****p ≤0.0001).

In order to further confirm that the observed effect of MH is independent of its LPS content, we administered MH i.p. in C3H/HeJ mice, which are genetically hyporesponsive to LPS due to their expression of a mutated, non-functional, TLR4 protein (20), and investigated the subsequent PEC response. The findings obtained using these TLR4-defective mice were quite similar to those observed in C57BL/6 mice (**Figure 6**). There was a substantial 2.4-fold increase in the number of PECs following MH administration compared to control mice injected with SC solution (**Figure 6A**). The majority (64%) of the PECs in MH-injected mice were myeloid cells (**Supplementary Figures 3A, H**). In contrast, the percentage of B1, B2 and NK cells was significantly decreased (**Supplementary Figures 3E–G**) in MH-treated mice, while T cells and dendritic cells exhibited insignificant alterations in their ratio within the PECs (**Supplementary Figures 3C, D**). The greatest change in the number of PECs occurred in the neutrophil subpopulation where their number increased from ~3000 cells in SC-treated mice to 1.0 x 10^6^ cells (~330-fold increase) in MH-treated C3H/HeJ mice (**Figures 6B–D**). These findings further confirm that the MH-induced recruitment of neutrophils into the peritoneal cavity is not mediated by any potential LPS component in MH and is independent of TLR4 recognition (**Figure 6**).

**Figure 7:**
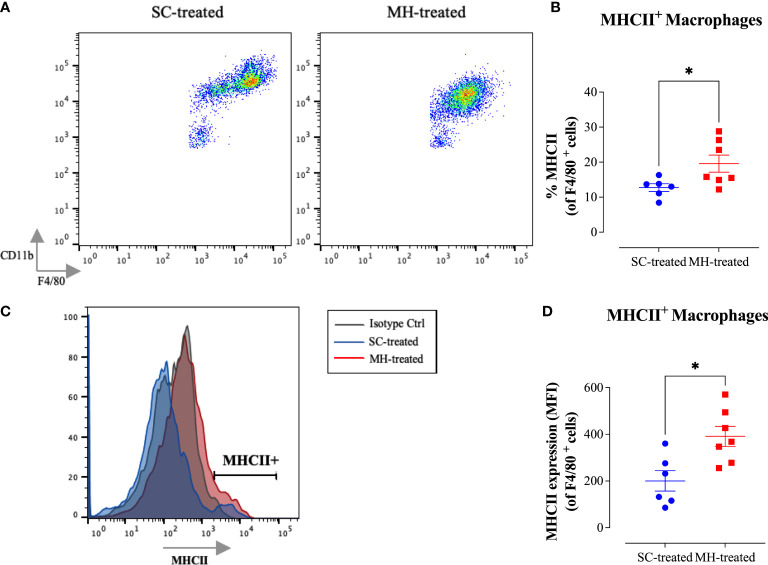
MH induces maturation of peritoneal myeloid cells in C3H/HeJ mice. **(A)** Representative dot plots showing F4/80^+^ CD11b^+^ cells (gated on macrophages) in SC-treated and MH-treated mice. **(B)** Representative flow cytometric histogram showing MHCII expression on F4/80^+^ macrophages of SC-treated and MH-treated mice. Grey histogram indicates staining with isotype-matched control antibody. **(C, D)** Quantification of the percentage **(C)** and median fluorescence intensity **(D)** of MHC class II^+^ macrophages in SC-treated and MH-treated groups. Asterisks denote statistically significant differences between the MH-treated group and the SC-treated groups. The values for individual mice in a group ± SEM are shown (SC-treated: n=6, MH-treated: n=7), pooled from 2 individual experiments. p values were calculated using the unpaired Student’s t-test (*p ≤ 0.05).

Further analysis of the peritoneal macrophage population indicated similar MH-induced alterations in their characteristics to those observed in C57BL/6 mice (**Figure 7**). This is highlighted by the decrease in cell surface expression of F4/80 and CD11b macrophage markers (**Figure 7A**) and a substantial increase in MHC class II protein expression on PEC macrophages (**Figures 7B–D**), resulting in a significant increase in the percentage of MHC class II-positive cells in this population (**Figure 7C**). It can be concluded that the observed PEC response following MH administration is independent of TLR4 recognition.

### Manuka honey-induced PECs response is dependent on MyD88-mediated signaling

The TLR family contains 12 members in the mouse and respond to many ligands (31). With the exception of TLR3, all other known TLRs are dependent on the MyD88 adaptor protein for their function (32). MyD88 is also an essential adaptor in the pro-inflammatory IL-1 and IL-18 receptor signaling pathways (33). The major role of the MyD88 protein is to link the triggering of TLRs, and IL-1/IL-18 receptors, by their respective cognate ligands to downstream activation of IL-1 receptor-associated kinases (IRAKs) and nuclear factor-kappa B (NF-kB). Given the major role MyD88 plays in inflammatory pathways, we investigated its potential involvement in MH-induced immune responses using mice genetically deficient in MyD88 protein (MyD88^-/-^ mice).

**Figure 8:**
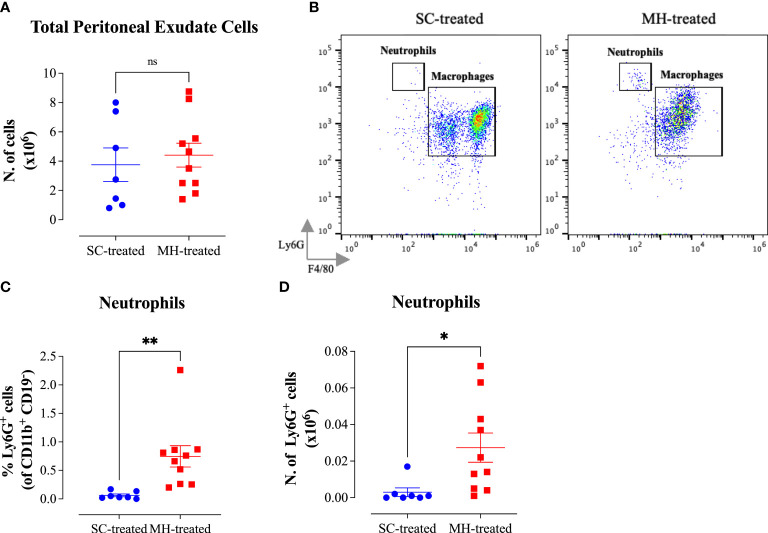
Impairment in the peritoneal response following MH administration in MyD88^-/-^ mice. **(A)** Total number of PECs following treatment with SC or MH. **(B)** Representative dot plots showing Ly6G^+^ neutrophils and F4/80^+^ macrophages within the PECs (gated on CD11b^+^ CD19^-^ CD11c^-^ cells). **(C, D)** Quantification of the percentage **(C)** and absolute number **(D)** of neutrophils (Ly6G^+^ cells) in the peritoneal cavity of MyD88^-/-^ mice following treatment with SC or MH. Asterisks denote statistically significant differences between the MH-treated and SC-treated groups. The values for individual mice (mean ± SEM) are shown (SC-treated: n=7; MH-treated: n=10), pooled from 2 independent experiments. *p* values were calculated using the unpaired Student’s t-test (ns; p >0.05, *p ≤0.05, **p ≤0.01).

In contrast to C57BL/6 and C3H/HeJ mouse strains, no significant infiltration into the peritoneal cavity was observed following MH administration in MyD88^-/-^ mice (**Figure 8A**). Moreover, the cellular changes in the PECs were relatively minor in comparison to what we observed in the other two mouse strains (**Supplementary Figures 4A–N**). Nevertheless, a small, but significant, increase in the percentage and absolute number of neutrophils was still observed following the treatment. The extent of the increase in neutrophils, however, was much smaller than what was observed in C57BL/6 and C3H/HeJ mice (**Figures 8B–D**). For example, within the PEC myeloid population, the % of neutrophils in MH-treated mice reached 11.5% and 14.6% in C57BL/6 (**Figure 3C**) and C3H/HeJ (**Figure 6C**) mice, respectively. In contrast, the % of neutrophils in MH-treated MyD88^-/-^ mice was only 0.75% (**Figure 8C**). Similar observations apply to the absolute number of neutrophils, where their mean counts were 0.5 x 10^6^ and 1.0 x10^6^ cells in MH-treated C57BL/6 (**Figure 3D**) and C3H/HeJ (**Figure 6D**) mice. However, their mean reached only 0.03 x 10^6^ cells in MH-treated MyD88^-/-^ mice (**Figure 8D**).

**Figure 9:**
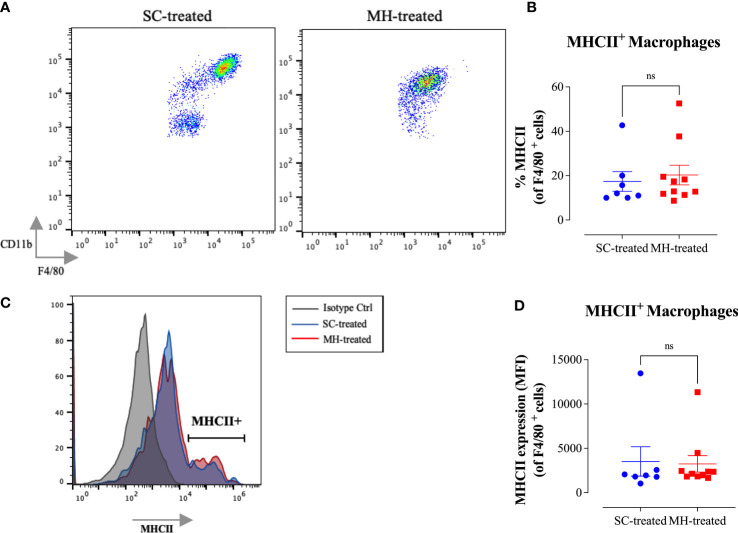
No evidence for myeloid cell maturation in the peritoneal cavity of MyD88^-/-^mice after MH injection. **(A)** Representative dot plots showing F4/80^+^ CD11b^+^ cells (gated on macrophages) in SC-treated and MH-treated mice. **(B)** Representative histograms showing MHC class II expression on F4/80^+^ macrophages of SC-treated and MH-treated mice. Grey histogram indicates staining with isotype-matched control antibody. **(C, D)** Quantification of the percentage **(C)** and median fluorescence intensity **(D)** of MHC class II^+^ macrophages in SC-treated and MH-treated groups. Asterisks denote statistically significant differences between the MH-treated and SC-treated groups. The values for individual mice (mean ± SEM) are shown (SC-treated: n=7; MH-treated: n=10), pooled from 2 independent experiments. *p* values were calculated using two-tailed Student’s t-test (ns; p > 0.05).

Finally, concerning the characterization of peritoneal macrophages, there was no evidence of functional maturation of these cells in MH-treated MyD88^-/-^ mice (**Figure 9**). Although there was a small decrease in cell surface expression of F4/80 and CD11b macrophage markers (**Figure 9A**) no alteration in MHC class II protein expression was noted (**Figures 9B–D**). Taken together, these findings suggest that the peritoneal response to MH administration is severely blunted in the absence of MyD88 expression.

## Discussion

Manuka honey is widely used as an alternative natural medicine for a wide range of disorders. In addition to its known antimicrobial (34), wound healing (35), and anti-cancer effects (17, 18, 23, 26), it has been shown to have immunomodulatory properties (9, 12, 36). In the present study, we aimed to characterize mechanistically the immunomodulatory potential of MH using both *in vitro* and *in vivo* approaches.

There are four major findings of the current study. First, MH is able to trigger a substantial level of activation of the pleiotropic cytokine TNF-α by macrophages. Second, MH induces the recruitment of neutrophils in an acute model of exposure through the induction of a chemokine response. Third, MH promotes the functional maturation of peritoneal macrophages, most likely *via* a cytokine-dependent mechanism. Fourth, these MH-mediated effects on neutrophils and macrophages involve MyD88-dependent signaling pathways.

The activation of TNF-α at the mRNA and protein levels in RAW 264.7 macrophages following MH-treatment confirms and extends previous studies which demonstrated the ability of several types of honey to stimulate the release of inflammatory cytokines from different monocytic cell lines, including MM6, THP-1, and U937 cells (9, 11, 36). In contrast to these findings, another study found no evidence of increased mRNA and protein levels of pro-inflammatory cytokines, including TNF-α, following exposure of RAW 264.7 cells to MH for 24 hours (12). In our study, we focused on using MH at a concentration of 1% w/v (equivalent to 10 mg/ml), a concentration previously shown to be non-toxic to cells (18, 26). The honey concentrations used in Afrin’s study (3 mg/ml and 8 mg/ml) are lower than the one used in our study. Thus, it is possible that the difference in the findings could be due to the variation in the MH concentrations used. However, it is worth noting that, in a previous study, a concentration of 0.5% w/v of MH was shown to induce TNF-α release from 2 different human monocytic cell lines, albeit at an order of magnitude lower than what we are reporting here (11). Another possible reason for the difference in our findings compared to Afrin’s study is the type of honey used. While we have used UMF 20+ MH throughout our study, the UMF of the MH used in Afrin’s study was not reported. Thus, differences in the concentrations of bioactive components in the various MH types could well be responsible for the different findings (37). It is important to note that the capacity of MH to induce the TNF-α response by macrophages plays an important role not only in boosting pro-inflammatory responses but could also contribute to its wound healing properties (11, 38, 39).

Several studies have attempted to identify the nature of the components within MH responsible for its immunostimulatory properties. Tonks and co-workers isolated a heat-labile, non-protein, polymyxin B-insensitive, 5.8-kDa component from MH that stimulated a human monocyte cell line to release TNF-α *via* a TLR4-dependent mechanism (10). Other studies demonstrated that a major protein component of honey and royal jelly, apalbumin-1, could induce the secretion of TNF-α by macrophages and keratinocytes (40, 41). However, it is not known how apalbumin-1 interacts with myeloid cells. Another group of investigators identified type II arabinogalactan proteins as the components in honey responsible for induction of TNF-α release by macrophages (11). These high molecular weight polysaccharide-protein complexes are widely found in plants and have been shown to possess immunostimulatory capacity (42–45). Moreover, there is evidence that type II arabinogalactans act on myeloid cells *via* TLR2 and TLR4 receptors (46). Thus, two of the components of honey identified so far to possess immunostimulatory activities on macrophages, namely arabinogalactan proteins and the 5.8 kDa component, act *via* interaction with different TLRs.

In addition to its capacity to induce cytokine secretion *in vitro*, i.p. administration of MH triggered a dramatic peritoneal response in normal mice. This response is highlighted by the substantial increase in the total number of peritoneal cells and the recruitment of neutrophils. Our findings are consistent with previous studies showing that i.p. administration of jungle honey could induce recruitment of neutrophils into the peritoneal cavity of C57BL/6 mice, which were then able to limit tumor growth within the same site (47). The observed recruitment of neutrophils is directed through the secretion of chemokines which are primarily involved in the recruitment of inflammatory cells into the sites of injury (48). In this regard, our present data confirm the ability of MH to upregulate the gene expression of CCL2 and CXCL2 chemokines by macrophages, two of the most potent chemoattractants for polymorphonuclear leukocytes (29). It is reasonable to suggest that the observed cell recruitment into the peritoneal cavity is initiated by the release of chemokines from peritoneal macrophages after stimulation by MH. Nonetheless, it would be important to demonstrate this directly by sorting the PECs and analyzing the alterations in their genetic signatures after exposure to MH.

In our efforts to study the mechanism underlying this response, we utilized two strains of mice with known defects in TLR signaling pathways. C3H/HeJ mice express a mutated, dysfunctional, TLR4 protein and hence are unable to respond to LPS (20). TLRs are known to play crucial roles in the innate immune system through their recognition of conserved pathogen-associated molecular patterns of diverse microbes. Increasingly, it is becoming clear that TLRs can also recognize endogenous as well as non-pathogen-associated ligands and, hence, play crucial roles not only in inflammation and host defense but also in maintaining homeostasis of the immune system (19, 49, 50). as well as in cancer development and response to therapy (51–53). Most TLRs utilize the MyD88 adaptor protein to link with downstream signaling pathways, leading to the induction of inflammatory cytokines, chemokines, type I interferon (IFN), and other mediators (32). Therefore, we also used MyD88-deficient mice to investigate the peritoneal response to MH. Our data clearly show that the peritoneal response to MH was evident in C3H/HeJ mice but not in MyD88^-/-^ mice. This suggests that TLR4 is not involved in MH-induced TNF-α activation and recruitment of neutrophils and further confirms that this response is not due to LPS. However, given the severely reduced response observed in the absence of MyD88 protein, other TLRs could be involved in the MH-triggered response. For example, since type II arabinogalactan proteins are known to signal through TLR2, it is possible that MH-initiated immunostimulatory response could be mediated through TLR2.

Our results also indicated some changes in the distribution of peritoneal macrophages following MH-treatment, characterized by a shift from CD11b^hi^ F4/80^hi^ cells to mostly CD11b^lo^ F4/80^lo^ cells. This phenomenon was previously described by Ghosn and co-workers who identified two macrophage subsets existing in the peritoneal cavity of adult mice (54). The first subset expresses elevated levels of CD11b and F4/80 markers and represents around 90% of the peritoneal macrophages in unstimulated mice. A second subset expresses low levels of CD11b and F4/80 markers and comprise the majority of peritoneal cells after LPS or thioglycolate stimulation (54). In our study, the phenotypic changes in peritoneal macrophages were accompanied by a significant increase in the expression of MHC class II molecules, suggesting the involvement of type I and/or type II interferons in this induction (55, 56). These phenotypic and functional alterations in peritoneal macrophages following MH administration were clearly evident in C57BL/6 and C3H/HeJ mice, but not in MyD88^-/-^ mice. This suggests the possibility that TLRs, other than TLR4, may be involved in this MH-induced activation. Further studies are needed to identify the actual MH moiety responsible for this immunostimulatory effect as well as the functional properties of these peritoneal macrophage populations.

Taken together, this study supports a role for MH as an immunomodulatory agent and identifies its capacity to induce immunostimulatory responses both *in vitro* and *in vivo*. The current findings implicate TLR signaling pathways in this process and highlight the potential use of MH, or its bioactive components, to boost immune responses, preventatively and therapeutically, in different disease settings, such as an adjunct treatment in cancer immunotherapy.

## Data availability statement

The original contributions presented in the study are included in the article/**Supplementary Material**. Further inquiries can be directed to the corresponding author.

## Ethics statement

The animal study was reviewed and approved by Institutional Animal Research Ethics Committee of the United Arab Emirates University.

## Author contributions

BA-R conceived the study and designed the experiments. RM, RN, YM, AA-S, GB, and BA-S performed experiments and data aquisition. RM, MF-C and BA-R analyzed experimental data. RM and BA-R wrote the manuscript. MF-C edited the manuscript. BA-R acquired the funding for the study and supervised the project. All authors contributed to the article and approved the submitted version.

## Funding

This work was supported by grants awarded to BA-R from Zayed Center for Health Sciences (#31R025) and UAEU Program for Advanced Research ((#G00002993), Office of Research and Sponsored Projects, United Arab Emirates University.​ RM was supported through a scholarship from the College of Graduate Studies, UAE University.

## Acknowledgments

The authors wish to acknowledge the United Arab Emirates University for supporting this project and the College of Graduate Studies for the PhD student scholarship awarded to RM.

## Conflict of interest

The authors declare that the research was conducted in the absence of any commercial or financial relationships that could be construed as a potential conflict of interest.

## Publisher’s note

All claims expressed in this article are solely those of the authors and do not necessarily represent those of their affiliated organizations, or those of the publisher, the editors and the reviewers. Any product that may be evaluated in this article, or claim that may be made by its manufacturer, is not guaranteed or endorsed by the publisher.

## Abbreviations

MH, manuka honey; SC, sugar control; TNF-α, tumor necrosis factor-alpha; IL-1β, interleukin-1β, IL-6, interleukin-6; iNOS, inducible nitric oxide synthase; IP-10, interferon gamma-induced protein 10; IFN-γ, interferon-gamma; MCP-1, monocyte chemoattractant protein-1; MIP, macrophage inflammatory protein; CXCL2, (C-X-C motif) ligand 2, CCL2, C-C motif ligand 2; CXCL10, C-X-C motif ligand 10; TLR4, toll-like receptor 4; TLR, toll-like receptor; MyD88, myeloid differentiation primary response 88; MHCII, major histocompatibility complex II; PECs, peritoneal exudate cells; MGO, methylglyoxal; UMF, unique manuka factor.
