# Supplementary material for: Characterization of immunomodulatory responses induced by manuka honey
Source: Front Immunol. 2022 Nov 2;13:1020574. doi: 10.3389/fimmu.2022.1020574 (PMC9670174; doi:10.3389/fimmu.2022.1020574)
Supplement: Supplementary file 1 [file DataSheet_1.pdf]

Supplementary Figures

Characterization of Immunomodulatory Responses Induced by Manuka Honey

Razan J. Masad[1], Rasha A. Nasser[1], Ghada Bashir[1], Yassir A. Mohamed[1], Ashraf Al-Sbiei[2], Besan H. Al-Saafeen[1], Maria J. Fernandez-Cabezudo[2], & Basel K. al-Ramadi[1,3]*

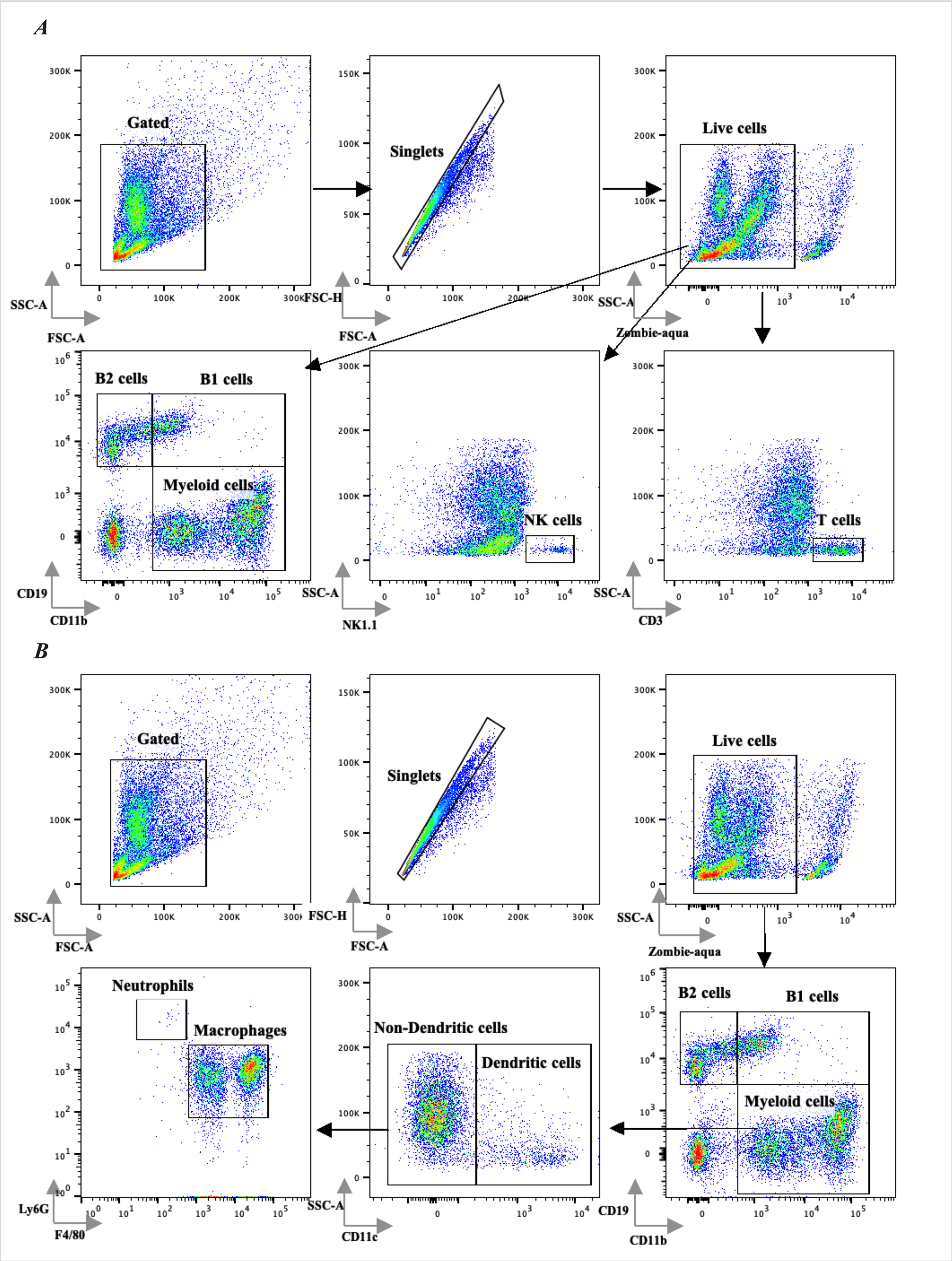

Supplementary figure 1. Gating strategy used for the identification of the major cellular populations in the peritoneal cavity of the mice. (A) Phenotype panel; single-cell suspensions were stained with different fluorophore-conjugated antibodies and analyzed by flow cytometry. Among the single cells, the live cells were selected for further analysis to identify B1 cells ($CD19^{+}$ $CD11b^{+}$), B2 cells ($CD19^{+}$ $CD11b^{-}$), myeloid cells ($CD19^{-}$ $CD11b^{+}$), T cells ($CD3^{+}$), and NK cells ($NK1.1^{+}$/ $CD49b^{+}$). (B) Myeloid panel; single-cell suspensions were stained with different fluorophore-conjugated antibodies and analyzed by flow cytometry. Among the single cells, the live cells were selected for further analysis to identify B1 cells ($CD19^{+}$ $CD11b^{+}$), B2 cells ($CD19^{+}$ $CD11b^{-}$), and myeloid cells ($CD19^{-}$ $CD11b^{+}$). Among the myeloid cells, the dendritic cells ($CD11c^{+}$), macrophages ($F4/80^{+}$), and neutrophils ($Ly6G^{+}$) were identified.

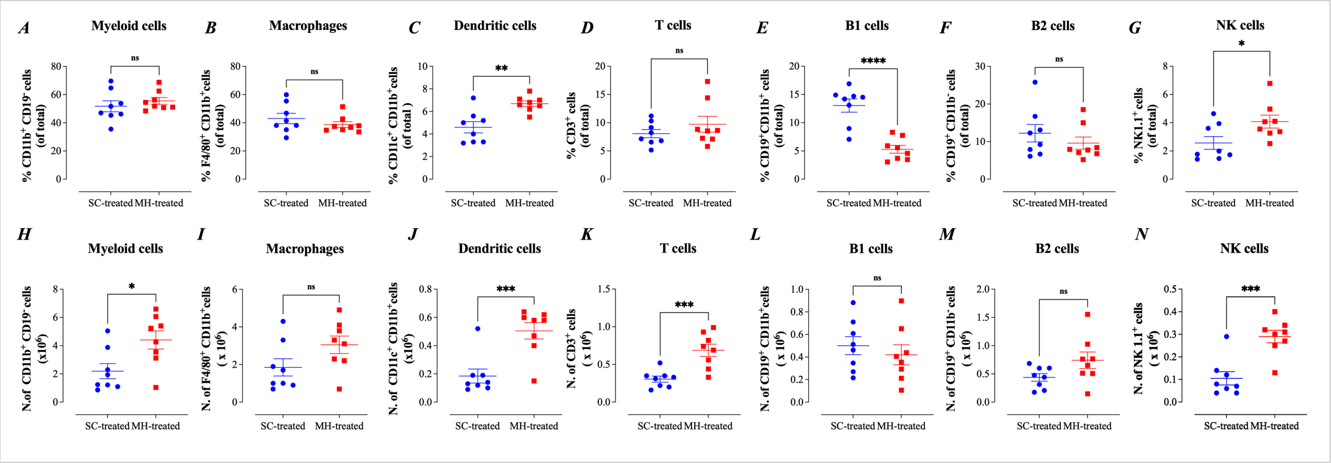

Supplementary figure 2. Evaluation of the lymphoid and myeloid cellular populations in the peritoneal cavity of C57BL/6 mice. (A-G) Cell percentages of PECs; myeloid cells (CD11b$^+$ CD19$^-$) (A), macrophages (F4/80$^+$ CD11b$^+$) (B), dendritic cells (CD11c$^+$ CD11b$^+$) (C), T cells (CD3$^+$) (D), B1 cells (CD19$^+$ CD11b$^+$) (E), B2 cells (CD19$^+$ CD11b$^-$) (F), and NK cells (NK 1.1$^+$) (G). (H-N) Total cell numbers of myeloid cells (CD11b$^+$ CD19$^-$) (H), macrophages (F4/80$^+$ CD11b$^+$) (I), dendritic cells (CD11c$^+$ CD11b$^+$) (J), T cells (CD3$^+$) (K), B1 cells (CD19$^+$ CD11b$^+$) (L), B2 cells (CD19$^+$ CD11b$^-$) (M), and NK cells (NK 1.1$^+$) (N). Asterisks denote statistically significant differences between the MH-treated and SC-treated groups. The values for individual mice in a group ± SEM are shown (SC-treated: n=8, MH-treated: n=8), pooled from 2 individual experiments. p values were calculated using the unpaired Student's t-test (ns: not significant, * $p \leq 0.05$, ** $p \leq 0.01$, *** $p \leq 0.001$, **** $p \leq 0.0001$).

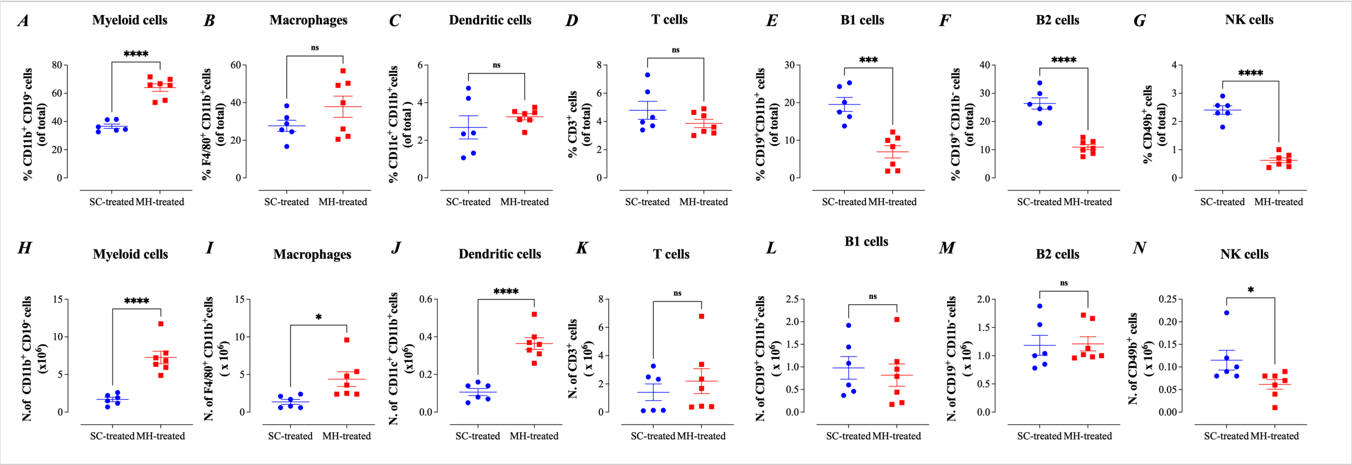

Supplementary figure 3. Evaluation of the lymphoid and myeloid cellular populations in the peritoneal cavity of C3H/HeJ mice. (A-G) Cell percentages of PECs; myeloid cells (CD11b$^+$ CD19$^-$) (A), macrophages (F4/80$^+$ CD11b$^+$) (B), dendritic cells (CD11c$^+$ CD11b$^+$) (C), T cells (CD3$^+$) (D), B1 cells (CD19$^+$ CD11b$^+$) (E), B2 cells (CD19$^+$ CD11b$^-$) (F), and NK cells (CD49b$^+$) (G). (H-N) Total cell numbers of myeloid cells (CD11b$^+$ CD19$^-$) (H), macrophages (F4/80$^+$ CD11b$^+$) (I), dendritic cells (CD11c$^+$ CD11b$^+$) (J), T cells (CD3$^+$) (K), B1 cells (CD19$^+$ CD11b$^+$) (L), B2 cells (CD19$^+$ CD11b$^-$) (M), and NK cells (CD49b$^+$) (N). Asterisks denote statistically significant differences between the MH-treated and SC-treated groups. The values for individual mice in a group ± SEM are shown (SC-treated: n=6, MH-treated: n=7), pooled from 2 individual experiments. p values were calculated using the unpaired Student's t-test (ns: not significant, * $p \leq 0.05$, *** $p \leq 0.001$, **** $p \leq 0.0001$).

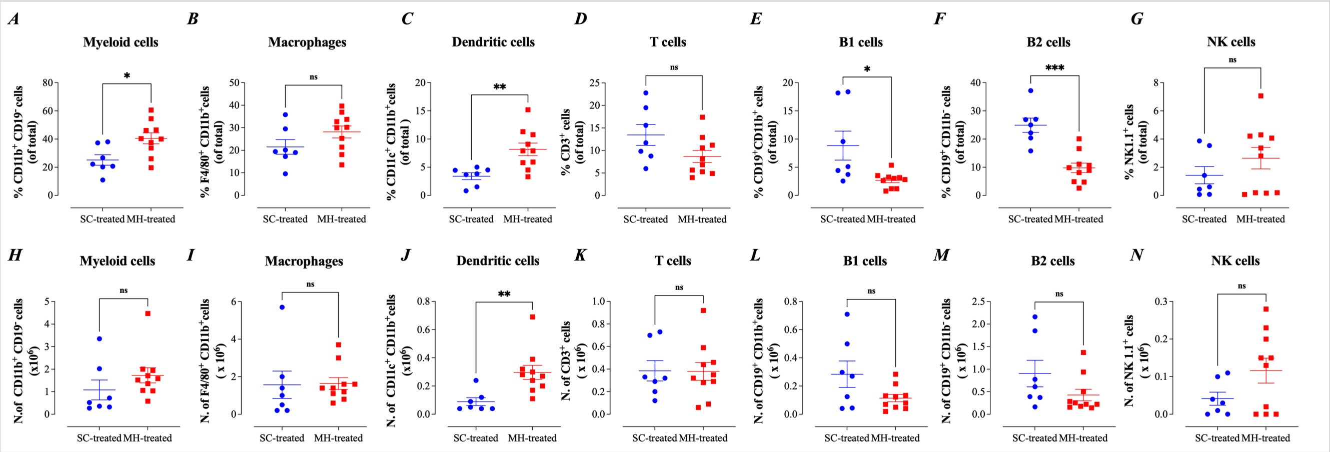

Supplementary figure 4. Evaluation of the lymphoid and myeloid cellular populations in the peritoneal cavity of MyD88$^{-/-}$ mice. (A-G) Cell percentages of PECs; myeloid cells (CD11b^{+} CD19^{-}) (A), macrophages (F4/80^{+} CD11b^{+}) (B), dendritic cells (CD11c^{+} CD11b^{+}) (C), T cells (CD3^{+}) (D), B1 cells (CD19^{+} CD11b^{+}) (E), B2 cells (CD19^{+} CD11b^{-}) (F), and NK cells (NK 1.1^{+}) (G). (H-N) Total cell numbers of myeloid cells (CD11b^{+} CD19^{-}) (H), macrophages (F4/80^{+} CD11b^{+}) (I), dendritic cells (CD11c^{+} CD11b^{+}) (J), T cells (CD3^{+}) (K), B1 cells (CD19^{+} CD11b^{+}) (L), B2 cells (CD19^{+} CD11b^{-}) (M), and NK cells (NK 1.1^{+}) (N). Asterisks denote statistically significant differences between the MH-treated and SC-treated groups. The values for individual mice in a group ± SEM are shown (SC-treated: n=7, MH-treated: n=10), pooled from 2 individual experiments. p values were calculated using the unpaired Student's t-test (ns: not significant, * $p \leq 0.05$, ** $p \leq 0.01$, *** $p \leq 0.001$).
